# Development of a CT image analysis-based scoring system to differentiate gastric schwannomas from gastrointestinal stromal tumors

**DOI:** 10.3389/fonc.2023.1057979

**Published:** 2023-06-28

**Authors:** Sheng Zhang, Zhiqi Yang, Xiaofeng Chen, Shuyan Su, Ruibin Huang, Liebin Huang, Yanyan Shen, Sihua Zhong, Zijie Zhong, Jiada Yang, Wansheng Long, Ruyao Zhuang, Jingqin Fang, Zhuozhi Dai, Xiangguang Chen

**Affiliations:** ^1^ Department of Radiology, Meizhou People’s Hospital, Meizhou, China; ^2^ Guangdong Provincial Key Laboratory of Precision Medicine and Clinical Translational Research of Hakka Population, Meizhou People’s Hospital, Meizhou, China; ^3^ Department of Radiology, First Affiliated Hospital of Shantou University Medical College, Shantou, China; ^4^ Department of Radiology, Jiangmen Central Hospital, Guangdong, China; ^5^ Department of Radiology, The First Affiliated Hospital of Huzhou University, Huzhou, Zhejiang, China; ^6^ Research Center Institute, United Imaging Healthcare, Shanghai, China; ^7^ Department of Radiology, Shantou Central Hospital, Shantou, Guangdong, China; ^8^ Department of Radiology, Daping Hospital, Army Medical University, Chongqing, China; ^9^ Department of Radiology, Sun Yat-sen Memorial Hospital, Sun Yat-sen University, Guangzhou, Guangdong, China

**Keywords:** stomach neoplasms, gastrointestinal stromal tumor, multidetector computed tomography, image processing, computer-assisted

## Abstract

**Purpose:**

To develop a point-based scoring system (PSS) based on contrast-enhanced computed tomography (CT) qualitative and quantitative features to differentiate gastric schwannomas (GSs) from gastrointestinal stromal tumors (GISTs).

**Methods:**

This retrospective study included 51 consecutive GS patients and 147 GIST patients. Clinical and CT features of the tumors were collected and compared. Univariate and multivariate logistic regression analyses using the stepwise forward method were used to determine the risk factors for GSs and create a PSS. Area under the receiver operating characteristic curve (AUC) analysis was performed to evaluate the diagnostic efficiency of PSS.

**Results:**

The CT attenuation value of tumors in venous phase images, tumor-to-spleen ratio in venous phase images, tumor location, growth pattern, and tumor surface ulceration were identified as predictors for GSs and were assigned scores based on the PSS. Within the PSS, GS prediction probability ranged from 0.60% to 100% and increased as the total risk scores increased. The AUC of PSS in differentiating GSs from GISTs was 0.915 (95% CI: 0.874–0.957) with a total cutoff score of 3.0, accuracy of 0.848, sensitivity of 0.843, and specificity of 0.850.

**Conclusions:**

The PSS of both qualitative and quantitative CT features can provide an easy tool for radiologists to successfully differentiate GS from GIST prior to surgery.

## Introduction

Gastrointestinal stromal tumors (GISTs) are the most common type of gastric mesenchymal tumors (GMTs) with potential malignancy (1–3). Gastric schwannomas (GSs) and leiomyomas, on the other hand, are different type of GMTs with a favorable prognosis (4, 5). There are variations in biological behavior, appropriate treatments, and prognoses between GISTs and benign mesenchymal tumors, such as GSs and leiomyomas. Asymptomatic GSs and leiomyomas can be followed up without surgery. However, surgical resection should be performed in GISTs patients with sizes larger than 2.0 cm due to the potential risk of metastasis. GISTs, GSs, and leiomyomas share similar clinical, imaging, and pathologic characteristics. However, GSs are commonly misdiagnosed as GISTs in comparison to leiomyomas due to moderate enhancement in imaging (1, 6). Therefore, it is imperative for clinicians to make an accurate distinction between GISTs and benign mesenchymal tumors, especially GSs, which is necessary for the development of personalized treatment plans and the ability to predict patient prognosis.

Endoscopy and endoscopic ultrasonography are useful tools for differentiating GSs from GISTs. However, endoscopy and endoscopic ultrasonography may have limitations in evaluating exophytic growth tumor, assessing lymph nodes, and relationship between tumor and adjacent structures. Contrast-enhanced computed tomography (CT) is another useful tool for distinguishing between GSs and GISTs, especially for exophytic growth tumors and tumors originating from the muscularis propria layer. Recently, several studies have focused on identifying the utility of CT qualitative or quantitative features to differentiate GSs from GISTs, and they found that several CT features, such as round contouring, exophytic or mixed growth patterns, homogeneous enhancement, and the CT attenuation value of tumors in arterial phase images, can be suggestive of GSs rather than GISTs (1, 2, 6, 7). Several of those CT features are overlapping with GIST and may be related to the tumor risk status as demonstrated by multiple studies (8, 9). However, considering the small sample size of GCs in these studies, the impact of these results in clinical practice may still be limited. A recent study by Wang et al. (5) showed that the developed model based on the combination of CT qualitative and quantitative features was useful for differentiating GSs from GISTs using a machine learning method. Their study included a relatively large cohort but did not consider potential confounding factors, such as different contrast media phases, and various CT systems, in the impact on CT quantitative features of tumors and did not validate the model using multicenter data. Furthermore, using the developed model might be time-consuming and difficult to apply in clinical practice. Therefore, in this multicenter study, we aimed to develop a point-based scoring system (PSS) to differentiate GSs from GISTs based on CT qualitative and quantitative features and to simplify its eventual clinical application.

## Methods

### Patient population

This retrospective study was approved by the institutional review board of Meizhou People’s Hospital and was performed in accordance with the Declaration of Helsinki, with the requirement for informed consent being waived. From August 2012 to March 2021, a total of 62 consecutive patients with GSs confirmed by postoperative histopathology and immunohistochemistry were enrolled in 6 independent institutions from 5 cities. Six GS patients were excluded because they did not have CT contrast-enhanced data, and three GS patients were excluded because they had no preoperative CT data. Finally, 51 GS patients were included in this study, and the mean age was 54.9 years (range: 23∼80 years).

Between August 2015 and December 2020, a control group of 147 consecutive GIST patients in the very low- or low-risk categories (hereinafter referred to as GISTs), which were confirmed by postoperative histopathology and immunohistochemistry from our institution, was recruited, and the mean age was 58.3 years (range: 22∼88 years). **Figure E1** in the **Supplementary Material** shows the patient recruitment pathway for the control group, along with the inclusion and exclusion criteria. This study only included patients with very low- or low-risk GIST, as these tumors are typically smaller in sizes, lack the potential of metastasis, and present with features resembling GSs, which can complicate differential diagnosis. In contrast, high-risk GISTs are easier to distinguish from other tumors due to their metastatic potential.

### CT protocol acquisition

Patients fasted for a minimum of 6 hours and were trained to hold their breath before the CT scan and were provided with 800∼1000 ml of water to achieve gastric distension. All patients underwent triphasic CT scanning, including an unenhanced scan and arterial phase and venous phase contrast-enhanced scans on different CT systems. We selected patient images that were acquired on various models of multirow spiral CT scans from GE, Siemens, Toshiba, and Philips scanners. The detailed acquisition parameters are summarized in **Table E1** in the **Supplementary Material**. The technical parameters were as follows:120/100 kVp, auto 200 mAs, slice thickness and slice interval 3.0/5.0 mm, matrix 512 × 512. All images were reconstructed into a 1.25 mm slice thickness with a slice interval of 1.25 mm.

### CT image analysis

The CT images were independently obtained by two senior radiologists (S.Z. and Z.Y., with more than 10 years of working experience). Both of them were blinded to the clinicopathological data.These findings were verified by another senior radiologist (X.C., with more than 15 years of working experience) to detect disagreements, if applicable. The CT qualitative features of tumors, including location (cardia and fundus, greater curvature of body, lesser curvature of body, or antrum), tumor growth patterns (endoluminal, exophytic, or mixed), heterogeneity (homogeneous vs. heterogeneous), contour (round or quasi-circular vs. lobulated), margin (well-defined vs. ill-defined), tumor surface ulceration (absent vs. present), intralesional hemorrhage (negative vs. positive), cystic change (negative vs. positive), necrosis (negative vs. positive), and calcification (negative vs. positive), were extracted and recorded. Endoluminal/mixed growth tumors were defined as tumors located within/across the margin of gastrointestinal structures, and exophytic growth tumors were defined as intracavitary tumors extending beyond the margin of the gastrointestinal structure profile (1). Tumor surface ulceration was defined as slit- or semi-elliptical-shaped lesions of gastric mucosa extending to the tumor (1). The descriptions of CT qualitative features are listed in **Table E2** in the **Supplementary Materials**.

The CT quantitative parameters were independently analyzed by the two aforementioned senior radiologists blinded to the clinicopathological data. The mean value of the two radiologists was utilized for the final analysis. By manually placing circular regions of interest (ROIs) of approximately 10.0–30.0 mm^2^ on the maximal section with the greatest enhancement areas of tumors in each phase, the CT attenuation values in Hounsfield units (HU) of tumors in the noncontrast, arterial phase and venous phase contrast-enhanced images were recorded as ${\text{Value}}_{\text{ TNON}}$ , ${\text{ Value}}_{\text{ TA}}$ , and ${\text{Value}}_{\text{ TV}}$ , respectively. Vessel structures, necrosis, calcification, ulceration, and cystic areas should be avoided in the ROIs.

Furthermore, we measured the CT attenuation values of the spleen and aorta in the venous phase images as a reference standard and then compared it with the CT attenuation value of the tumors to eradicate confounding factors such as different patient cohorts with different contrast media phases, and various CT systems on the impact in the measurement of CT attenuation value of tumors (10). The same size of ROI was placed in the homogeneous spleen parenchyma on the greatest cross-section of the spleen to measure the CT attenuation value of the spleen (recorded as ${\text{Value}}_{\text{ SV}}$ ). Next, another ROI of the same size was placed in the same site of the spleen on the aorta to generate the aorta CT attenuation value (recorded as ${\text{Value}}_{\text{ AV}}$ ). All measurements were performed three times, and the mean results were recorded for further analysis. **Figure E2** in the **Supplementary Materials** is one example of the evaluation of CT features in venous phase contrast-enhanced images from a patient with a gastrointestinal stromal tumor.

To verify the reproducibility of CT quantitative and qualitative features, the determination of all features was repeated 3 weeks later by the same radiologist in the same way in a random order for all patients (11).

With Value _TNON_, Value _SV_, and Value _AV_ as references, the CT attenuation difference of tumors between arterial/venous phase and noncontrast images ( ${△}_{\text{A}}$ and ${△}_{\text{V}}$ ), attenuation difference between tumor and spleen ( ${△}_{\text{T}-\text{S}}$ ), and between tumor and aorta ( ${△}_{\text{T}-\text{A}}$ ), tumor contrast enhancement ratio (CER), tumor-to-spleen ratio (TSR), and tumor-to-aorta ratio (TAR) were calculated according to the following formulae:


(1) $${△}_{\text{A}/\text{V}}\;={\text{Value}}_{\text{ TA}/\text{TV}}-{\text{Value}}_{\text{ TNON }}$$

(2) $${△}_{\text{T}-\text{S}}\;={\text{Value}}_{\text{ TV}}-{\text{Value}}_{\text{ SV}}$$

(3) $${△}_{\text{T}-\text{A}}\;={\text{Value}}_{\text{ TV}}-{\text{Value}}_{\text{ AV}}$$

(4) $${\text{CER}}_{\text{ A}/\text{V}}={\text{Value}}_{\text{ TA}/\text{TV}}/{\text{Value}}_{\text{ TNON}}$$

(5) $${\text{CER}}_{\text{T}-\text{S}}={△}_{\text{T}-\text{S}}/{\text{Value}}_{\text{SV}}$$

(6) $${\text{CER}}_{\text{T}-\text{A}}={△}_{\text{T}-\text{A}}/{\text{Value}}_{\text{AV}}$$

(7) $$\text{TSR}={\text{Value}}_{\text{ TV}}/{\text{Value}}_{\text{ SV}}$$

(8) $$\text{TAR}={\text{Value}}_{\text{ TV}}/{\text{Value}}_{\text{ AV}}$$

where T indicates tumor, A and V indicate arterial phase and venous phase contrast-enhanced images, T-S indicates tumor to spleen, and T-A indicates tumor to aorta.

### Development of a point-based scoring system

The odds ratio (OR) with the corresponding 95% confidence interval (CI) for all variables was calculated using univariate and multivariate logistic regression analysis, according to the stepwise forward method, to determine the risk factors significantly associated with GSs. For each significant variable with a *P* value less than 0.05, a corresponding regression coefficient was obtained. The points for each significant variable were assigned according to the regression coefficients from the multivariate logistic regression model and rounded to the nearest integer. Next, the points were adapted from receiver operating characteristic (ROC) analysis to achieve the best discriminatory power of GSs. Finally, a point-based scoring system (PSS) was constructed using the method described by Sullivan et al. (12). ROC analysis was performed to evaluate the diagnostic efficiency of PSS and a logistic regression model. The area under the curve (AUC), accuracy, sensitivity, and specificity were calculated.

### Statistical analysis

R version 3.6.4 was used for statistical analysis. Quantitative variables with a normal distribution are presented as the mean ± standard deviation, and quantitative variables with an abnormal distribution are presented as the median with interquartile range in parentheses, while qualitative variables are shown as counts with percentages. For quantitative and qualitative imaging features, we used interclass correlation coefficients (ICCs) and Cohen’s kappa to analyze the consistency of the two radiologists (13). The CT imaging features were compared between GSs and GISTs using the chi-squared test (for categorical variables), Student’s *t*-test (for continuous variables with normal distribution), Mann–Whitney U test (for continuous variables with abnormal distribution), and Kruskal–Wallis H test (for ordinal variables). A *P*< 0.05 was considered significant. The AUCs of the logistic regression model and PSS were compared using the Delong test.

## Results

### Patient characteristics

A total of 198 patients, including 51 with GSs and 147 with GISTs, were reviewed in this study. The CT features of patients with GSs and GISTs are shown in **Table 1**. For all quantitative features, the maximum diameter, minimum diameter, and CT attenuation value of tumors in the venous phase images ( ${\text{Value}}_{\text{ TV}}$ ) were significantly higher in the GS group than in the GIST group (all *P*< 0.05). Using the nonenhanced CT attenuation value of tumors ( ${\text{Value}}_{\text{ TNON}}$ ) as a reference, the CT attenuation difference of tumors between venous phase and noncontrast images ( ${△}_{\text{V}}$ ) was higher in the GS group than in the GIST group (*P*<0.05). Using the CT attenuation value of the spleen in the venous phase ( ${\text{Value}}_{\text{ SV}}$ ) as a reference, ${△}_{\text{T}-\text{S}}$ and ${\text{CER}}_{\text{ T}-\text{S }}$ were lower in the GS group than in the GIST group (all *P*<0.001), while the TSR was higher in the GS group than in the GIST group (*P*<0.001). Similar results were observed in the $\;{△}_{\text{T}-\text{A}}$ , ${\text{ CER}}_{\text{ T}-\text{A }}$ and TAR (all *P*<0.001) with regard to the CT attenuation value of the aorta in the venous phase ( ${\text{Value}}_{\text{ AV}}$ ).

**Table 1:** CT features compared to gastric schwannoma and gastrointestinal stromal tumor patients.

Quantitative features	GISTs (*n*=147)	GSs (*n*=51)	*P value*	OR (95% CI)	*P^d^*
Age (years) ^#^	58.30 ± 11.16	54.92 ± 12.32	0.071	0.98 (0.95–1.00)	0.074
Maximum diameter (cm)	1.75 (1.15,2.90)	3.15 (1.90,4.70)	**<0.001**	1.67 (1.34–2.07)	**<0.001**
Minimum diameter (cm)	1.35 (0.81,2.41)	2.45 (1.51,3.85)	**<0.001**	1.56 (1.25–1.96)	**<0.001**
**Value_TNON_**	34.5 (29.0,41.0)	35.50 (31.5,39.0)	0.698	0.99 (0.96–1.03)	0.757
**Value_TA_**	51.5 (44.0,57.5)	56.0 (47.5,63.7)	0.061	1.01 (0.99–1.04)	0.427
**Value_TV_**	69.0 (59.0,82.0)	80.0 (64.0,86.5)	**0.015**	1.01 (0.99–1.03)	0.118
**Value_AV_**	154.0 (141.5,175.5)	139.0 (125.0,164.7)	**<0.001**	0.98 (0.97–0.99)	**0.006**
**Value_SV_**	127.0 (114.5,139.5)	110.5 (97.0,128.0)	**<0.001**	0.96 (0.95–0.98)	**<0.001**
**Δ_A_**	15.5 (10.0,23.0)	19.00 (13.5,27.2)	0.057	1.02 (0.99–1.05)	0.229
**Δ_V_**	33.5 (23.5,45.5)	41.9 (32.3,52.0)	**0.012**	1.02 (1.00–1.04)	0.067
**Δ_T-S_**	-57.0 (-71.5, -43.5)	-39.3 (-52. 0, -24.1)	**<0.001**	1.04 (1.02–1.05)	**<0.001**
**Δ_T-A_** ^#^	-87.14 ± 26.67	-69.15 ± 31.07	**<0.001**	1.03 (1.01–1.04)	**<0.001**
**CER_A_**	1.47 (1.24,1.79)	1.56 (1.37,1.78)	0.214	1.29 (0.60–2.81)	0.515
**CER_V_**	1.91 (1.64,2.46)	2.26 (1.80,2.55)	0.062	1.28 (0.82–1.98)	0.272
**CER_T-S_**	0.45 (0.37,0.54)	0.35 (0.26,0.43)	**<0.001**	0.007 (0.001–0.064)	**<0.001**
**CER_T-A_**	0.56 (0.49,0.62)	0.48 (0.38,0.53)	**<0.001**	0.006 (0.000–0.085)	**<0.001**
TSR	0.55 (0.46,0.63)	0.65 (0.58,0.77)	**<0.001**	70.56 (9.88–503.7)	**<0.001**
TAR	0.44 (0.38,0.51)	0.52 (0.47,0.62)	**<0.001**	166.1 (11.8–2338.9)	**<0.001**
Qualitative features					
Sex^*^			**<0.001**		
Male	60 (40.82%)	14 (27.45%)		–	–
Female	87 (59.18%)	37 (72.55%)		1.82 (0.91–3.66)	0.092
Tumor location^*^			**<0.001**		
Cardia and Fundus	88 (59.86%)	3 (5.88%)		–	–
Greater curvature	23 (15.65%)	25 (49.02%)		31.88 (8.84–115.0)	**<0.001**
Lesser curvature	32 (21.77%)	14 (27.45%)		12.83 (3.46–47.61)	**<0.001**
Antrum	4 (2.72%)	9 (17.65%)		66.00 (12.72–342.5)	**<0.001**
Growth pattern^*^			**<0.001**		
Endoluminal	112 (76.19%)	9 (17.65%)		–	–
Exophytic	13 (8.84%)	24 (47.06%)		22.97 (8.82–59.85)	**<0.001**
Mixed	22 (14.97%)	18 (35.29%)		10.18 (4.05–25.59)	**<0.001**
Heterogeneity^*^			0.298		
Homogeneous	98 (66.67%)	38 (74.51%)		–	–
Heterogeneous	49 (33.33%)	13 (25.49%)		0.68 (0.33-1.40)	0.300
Contour^*^			0.933		
Round/Quasi-circular	136 (92.52)	47 (91.56%)		–	–
Lobulated	11 (7.48%)	4 (7.84%)		1.05 (0.32–3.35)	0.933
Margin^*^			0.163		
Well-defined	146 (99.32%)	49 (96.08%)		–	–
Ill-defined	1 (0.68%)	2 (3.92%)		5.96 (0.53–67.16)	0.149
Tumor surface ulceration			**0.003**		
Absent	140 (95.24%)	41 (80.39%)		–	–
Present	7 (4.76%)	10 (19.61%)		4.88 (1.75–13.62)	**0.002**
Intralesional hemorrhage^*^			0.574		
Negative	143 (97.28%)	51 (100.0%)		–	–
Positive	4 (2.72%)	0 (0.00%)		0	0.999
Cystic change^*^			0.319		
Negative	134 (91.16%)	44 (86.27%)		–	–
Positive	13 (8.84%)	7 (13.73%)		1.64 (0.62–4.37)	0.323
Necrosis^*^			0.491		
Negative	132 (89.80%)	44 (86.27%)		–	–
Positive	15 (10.20%)	7 (13.73%)		1.40 (0.54–3.66)	0.492
Calcification^*^			0.745		
Negative	118 (80.27%)	42 (82.35%)		–	–
Positive	29 (19.73%)	9 (17.65%)		0.87 (0.38–1.99)	0.745

^*^Results are counts with the corresponding ratio in parentheses, Bold values represent results that are statistically significant. ^#^Results are mean value with standard deviation, and the remainder results are median with interquartile range in parentheses. **VALUE_TNON_**, **VALUE_TA_** and **VALUE_TV_** indicate the CT attenuation values of tumors in the noncontrast, arterial phase and venous phase images, respectively. **Δ_A_** indicates the CT attenuation difference of tumors between arterial phase and noncontrast images. **Δ_V_** indicates the CT attenuation difference of tumors between venous phase and noncontrast images. **Δ_T-S_** indicates the CT attenuation difference between tumor and spleen in the venous phase contrast-enhanced images. **Δ_T-A_** indicates the CT attenuation difference between the tumor and aorta in the venous phase images. **CER_A_** and **CER_V_** indicate tumor contrast enhancement ratios in the arterial phase and venous phase images, respectively. TSR indicates tumor-to-spleen enhancement ratio in the venous phase images. TAR indicates tumor-to-aorta ratio in the venous phase images. CER, Tumor contrast-enhancement ratio; TSR, Tumor-to-spleen ratio; TAR, Tumor-to-aorta ratio.

For all qualitative features, the most common gastric site of GSs was the greater curvature of the body (49.02%), and most GSs occurred in female patients (72.55%) compared with GIST patients. In addition, compared with GIST patients, most GSs tended toward exophytic (47.06%) or mixed (35.29%) growth patterns and had a higher incidence rate of tumor surface ulceration (19.61%).

### Interobserver consistency of CT quantitative and qualitative features

The Kappa values for location, growth pattern, heterogeneity, margin, surface ulceration, contour, intralesional hemorrhag, cystic change, necrosis, and calcification were 1.000, 0.903, 0.908,1.000,0.901,0.921,0. 962,0.991,0.974,and 0.908, respectively. The ICC values for quantitative features including maximum tumor diameter, minimum tumor diameter, ${\text{Value}}_{\text{ TNON}}$ , ${\text{Value}}_{\text{ TA}}$ , ${\text{Value}}_{\text{ TV}}$ , ${\text{Value}}_{\text{ SV}}$ , and ${\text{Value}}_{\text{ AV}}$ were 0.999 (95% CI: 0.999–0.999), 0.918 (95% CI: 0.893–0.938), 0.980 (95% CI: 0.970–0.986), 0.983 (95% CI: 0.977–0.988), 0.988 (95% CI: 0.984–0.991), 0.885 (95% CI: 0.851–0.912), and 0.996 (95% CI: 0.995–0.997), respectively.

### Univariate and multivariate analysis for factors predicting gastric schwannoma

Univariate logistic regression analysis (**Table 1**) showed that maximum diameter (OR=1.67, *P*<0.001), minimum diameter (OR=1.56, *P*<0.001), ${\text{Value}}_{\text{ AV}}$ (OR=0.98, *P*=0.006), ${\text{Value}}_{\text{ SV}}$ (OR=0.96, *P*<0.001), ${△}_{\text{T}-\text{S}}$ (OR=1.04, *P*<0.001), $\;{△}_{\text{T}-\text{A}}$ (OR=1.03, *P*<0.001), ${\text{CER}}_{\text{ T}-\text{S}}$ (OR=0.007, *P*<0.001), ${\text{ CER}}_{\text{ T}-\text{A}}$ (OR=0.006, *P*<0.001), TSR (OR=70.56, *P*<0.001), TAR (OR=166.1, *P*<0.001), tumor location in the greater curvature (OR=31.88, *P*<0.001), tumor location in the lesser curvature (OR=12.83, *P*<0.001), tumor location in the antrum (OR=66.00, *P*<0.001), exophytic growth pattern (OR=22.97, *P*<0.001), mixed growth pattern (OR=10.18, *P*<0.001), and tumor surface ulceration (OR=4.88, *P*=0.002) were associated with GSs.

Multivariate logistic regression analysis in the **Supplementary Materials** (**Table E3**) revealed that ${\text{Value}}_{\text{ TV}}$ (adjusted OR=0.96, *P*=0.042), TSR (adjusted OR=840.5, *P*=0.001), tumor location in the greater curvature (OR=15.33, *P*=0.001), tumor location in the lesser curvature (OR=5.53, *P*=0.038), tumor location in the antrum (OR=46.63, *P*<0.001), exophytic growth pattern (OR=17.75, *P*<0.001), mixed growth pattern (OR=6.33, *P*=0.002), and tumor surface ulceration (OR=6.83, *P*=0.011) were independently associated with GSs.

ROC curves were generated based on the prediction probability of the regression equation using the above variables. The combination logistic regression model that incorporated ${\text{Value}}_{\text{ TV}}$ , TSR, tumor location, growth pattern, and tumor surface ulceration yielded a maximum AUC of 0.929 (95% CI: 0.893–0.965), with an accuracy of 0.859, sensitivity of 0.922, and specificity of 0.837 (**Table 2**).

**Table 2:** Performance of the individualized prediction models.

Variables	Cutoff	AUC (95% CI)	Accuracy	Sensitivity	Specificity
Value_TV_	72.25	0.614 (0.526–0.703)	0.621	0.667	0.605
TSR	0.589	0.739 (0.663–0.815)	0.682	0.745	0.660
Location	2	0.761 (0.692–0.829)	0.687	0.941	0.599
Growth pattern	2	0.773 (0.699–0.847)	0.778	0.824	0.762
Surface ulceration	1	0.574 (0.478–0.670)	0.758	0.196	0.952
Combination	0.290	0.929 (0.893–0.965)	0.859	0.922	0.837

Value_TV_ indicates the CT attenuation value of tumor in the venous phase contrast enhanced images, TSR indicates tumor-to-spleen ratio in the venous phase images. “Combination” indicates the predicted model based on the combination of Value_TV_, TSR, tumor location, growth pattern, and tumor surface ulceration.

### Development of a point-based scoring system for predicting gastric schwannomas

The point-based scoring system (PSS) was created based on the results of a multivariate logistic regression analysis. Five variables were assigned scores for the final prediction rule based on their β-coefficient (**Table 3**): ${\text{Value}}_{\text{ TV}}$ [≥72.25 HU (0.0 points),<72.25 HU (1.0 points)], TSR[<0.589 (0.0 points), ≥0.589 (1.0 points)], tumor location [cardia and fundus (0.0 points), lesser curvature (1.0 points), greater curvature (2.0 points), and antrum (3.0 points)], growth patterns [endoluminal (0 points), mixed (1.0 points), exophytic (2.0 points)], tumor surface ulceration [absent (0 points), present (1.0 points)].

**Table 3:** Proposed point-based scoring system for predicting gastric schwannoma.

Variables	*β*-Coefficient	Points
Value_TV_		
≥72.25 HU	–	0
<72.25 HU	-0.038	-1.0
TSR		
<0.589	–	0
≥0.589	6.734	1.0
Tumor Location		
Cardia and fundus	—	0
Lesser curvature	1.711	1.0
Greater curvature	2.729	2.0
Antrum	3.842	3.0
Growth pattern		
Endoluminal	—	0
Mixed	1.845	1.0
Exophytic	2.876	2.0
Tumor surface ulceration		
Absent	—	0
Present	1.921	1.0

Value_TV_ indicates the CT attenuation value of tumor in the venous phase contrast-enhanced images. TSR indicates tumor-to-spleen ratio in the venous phase contrast-enhanced images.

**Figure 1:**
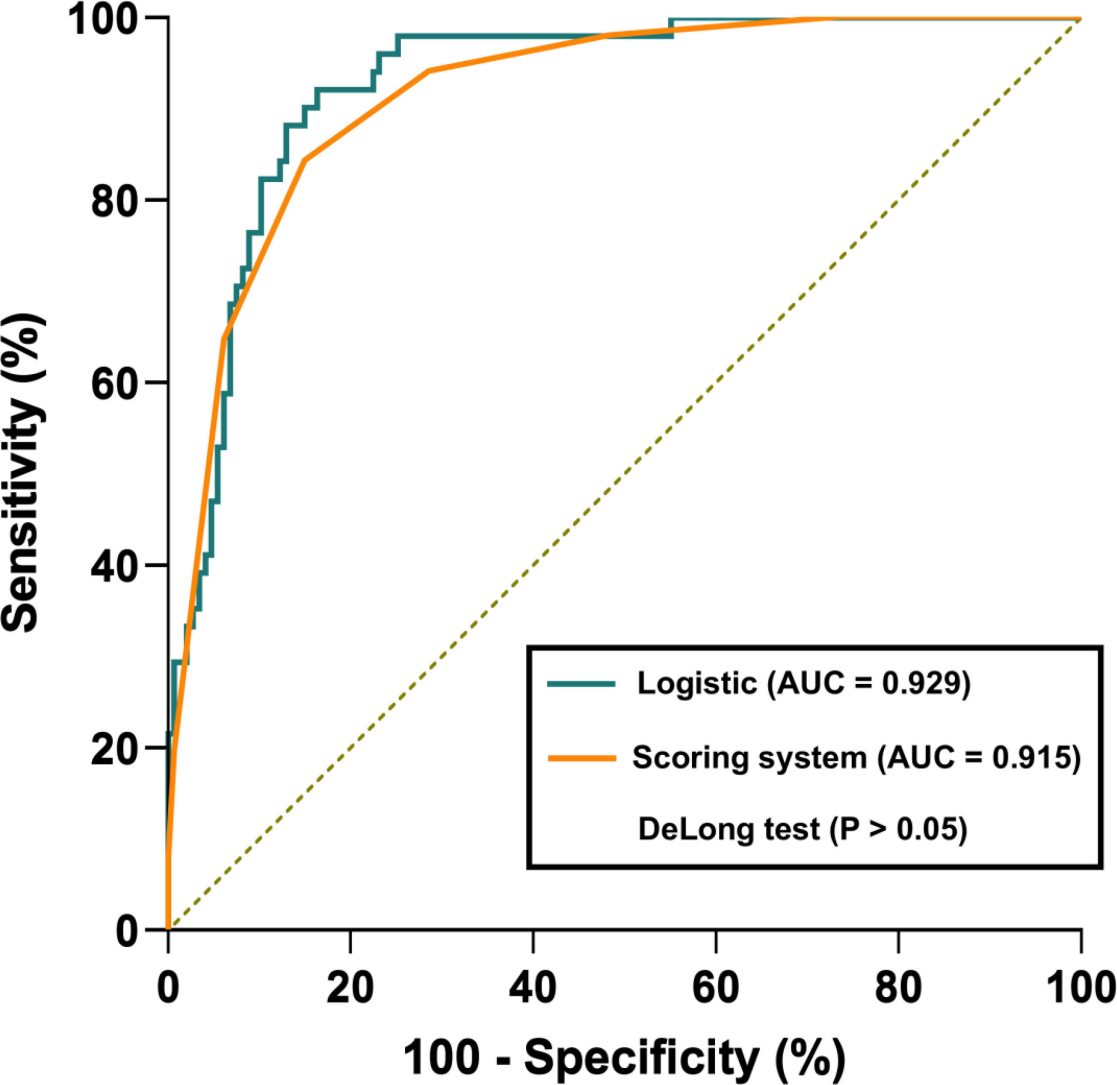
Receiver operator characteristic (ROC) curve comparison between the logistic regression model and point-based scoring system. The logistic regression model and point-based scoring system achieved an AUC of 0.929 and 0.915, respectively. However, there were no significant AUC differences between the logistic regression model and the scoring system (*P>*0.05).

**Figure 2:**
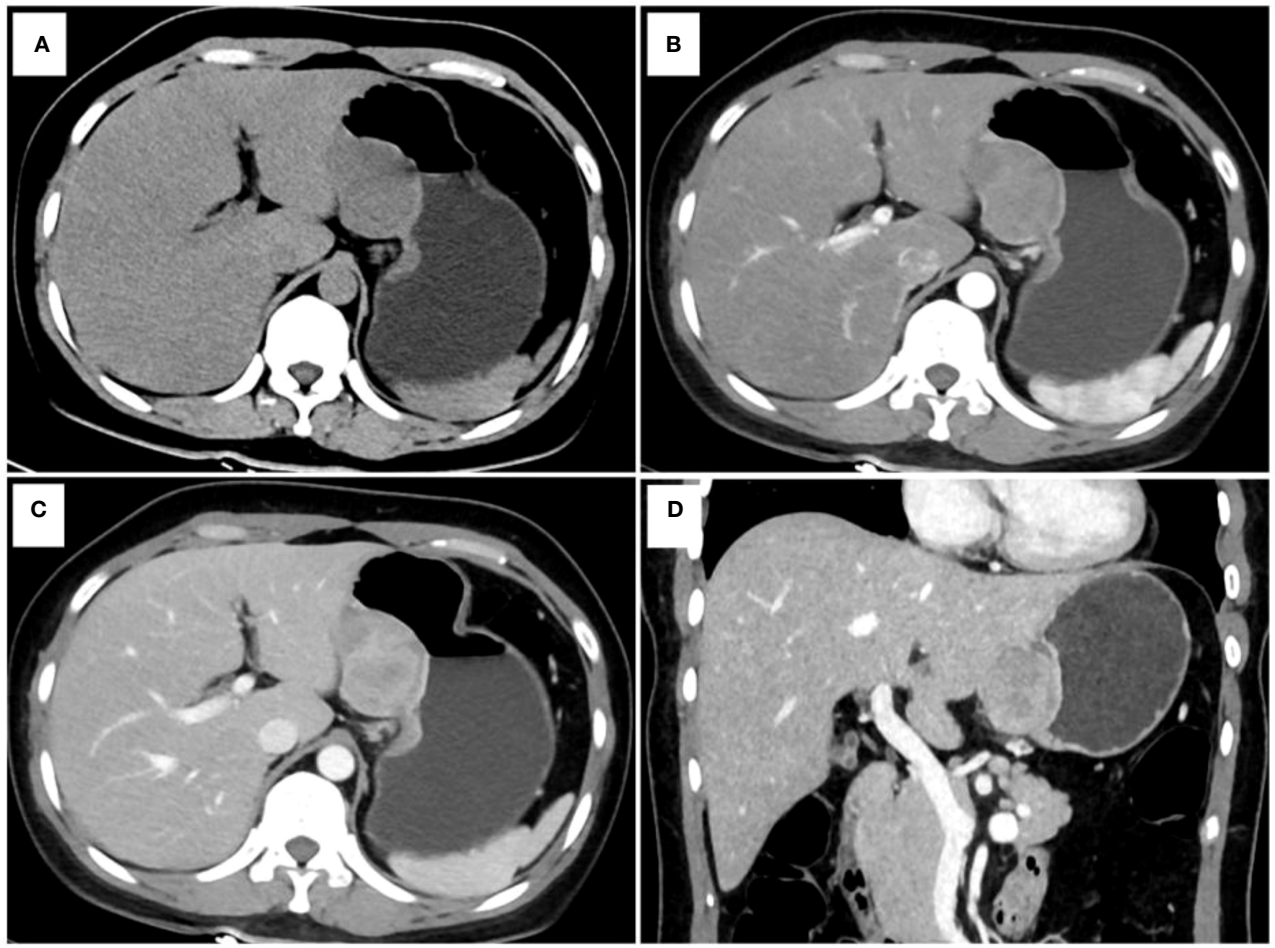
One example of a point-based scoring system in use. CT examination of patient 1, including axial unenhanced **(A)**, arterial phase image **(B)**, venous phase image **(C)**, and coronal venous phase image **(D)**, showed an exophytic growth pattern lesion in the lesser curvature of the gastric body without tumor surface ulceration. The CT attenuation value of the tumor in the venous phase images (Value_TV_) and the tumor-to-spleen ratio (TSR) were 119 HU and 0.84, respectively. The total risk score of gastric schwannoma (GS) assessed by the point-based scoring system (PSS) was 4 points, with a probability of 73.33%. Finally, the tumor was confirmed as GS by histopathology.

**Figure 3:**
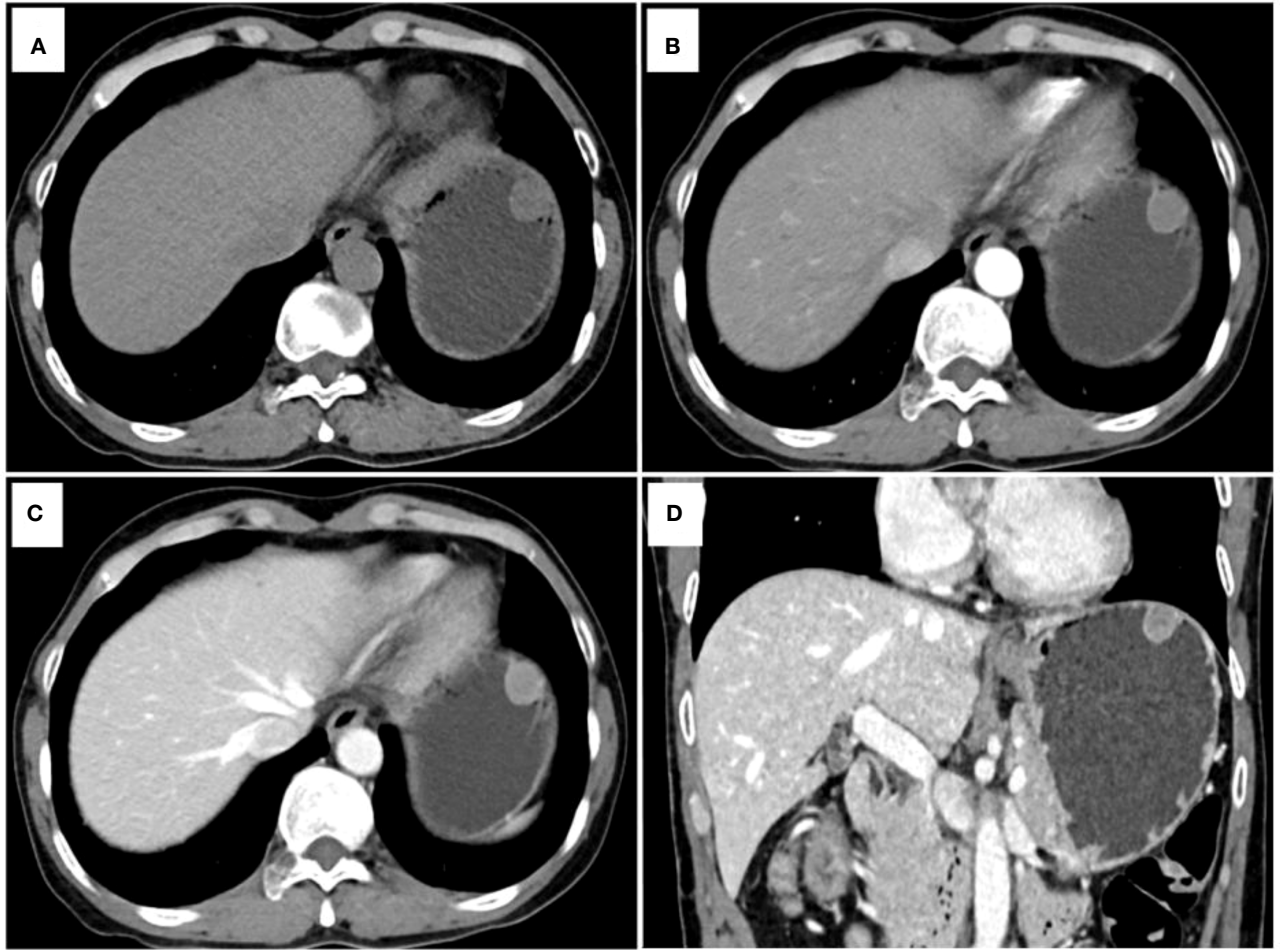
Another example of a point-based scoring system in use. CT examination of patient 2, including axial unenhanced **(A)**, arterial phase image **(B)**, venous phase image **(C)**, and coronal venous phase image **(D)**, showed an endoluminal growth pattern lesion in the gastric fundus without tumor surface ulceration. The CT attenuation value of the tumor in the venous phase images (Value_TV_) and the tumor-to-spleen ratio (TSR) were 79 HU and 0.54, respectively. The total risk score of gastric schwannoma (GS) was calculated to be 0 points with a low probability of 2.00% in the point-based scoring system (PSS). Pathology reports showed that the tumor was a gastrointestinal stromal tumor (GIST).

**Table 4** presents the prediction probability of GSs according to the PSS. The GS prediction probability ranged from 0.60% to 100% and increased as the total risk scores increased. The accuracy, sensitivity, and specificity were almost optimized when the critical value of total points for PSS was 3.0. Therefore, a total of 3.0 points was defined as the cutoff value between GSs and GISTs. The proposed PSS achieved an AUC of 0.915 (95% CI: 0.874–0.957), with an accuracy of 0.848, sensitivity of 0.843, and specificity of 0.850. There were no significant AUC differences between the logistic regression model and the scoring system (*P*=0.303) (**Figure 1**). Examples of point-based scoring systems in use are shown in **Figures 2** and **3**.

**Table 4:** Gastric schwannoma prediction probability according to the point-based scoring system.

Total risk scores	Probability	Accuracy	Sensitivity	Specificity
≥-1	≥0.60%	0.258	1.000	0.000
≥0	≥2.00%	0.455	1.000	0.265
≥1	≥6.50%	0.636	0.980	0.517
≥2	≥19.15%	0.773	0.941	0.714
≥3	≥44.66%	0.848	0.843	0.850
≥4	≥73.33%	0.864	0.647	0.939
≥5	≥90.36%	0.788	0.196	0.993
≥6	≥96.96%	0.763	0.078	1.000
≥7	≥100%	0.742	0.000	1.000

## Discussion

Differentiation of gastric schwannomas (GSs) from gastrointestinal stromal tumor (GISTs) has important clinical significance for patient treatment planning and prognosis. Previous studies have already evaluated different CT features from GISTs and submucosal tumors, but little attention has been paid to the difference between GSs and GISTs (2, 3, 14). Thus, in this study, an easy-use tool based on a point-based scoring system (PSS) was built to differentiate GSs from GISTs using CT quantitative and qualitative imaging features and was assessed for eventual clinical application. This PSS incorporated five predictors for GSs, including the CT attenuation value of tumors in venous phase images (Value_TV_), tumor-to-spleen ratio (TSR) in venous phase images, tumor location, growth pattern, and tumor surface ulceration, and achieved satisfactory diagnostic performance with an AUC of 0.915 for distinguishing GSs from GISTs. Due to the limitations of endoscopy and endoscopic ultrasonography for accurately diagnosing gastric tumors originating from the muscularis propria layer and exophytic growth tumors, this PSS can complement the current diagnostic path for gastric tumors originating from the muscularis propria layer and exophytic growth tumors.

Several studies have investigated the diagnostic performance for the identification of GSs and GISTs (1, 2, 5, 6). To the best of our knowledge, this was the first study to predict GSs in patients with GMTs using a developed PSS based on CT quantitative and qualitative imaging features. Since GSs can be easily misdiagnosed as GISTs, CT quantitative and qualitative features, such as changes in tumor enhancement in different contrast media phases, tumor location, and anatomical features, should be taken into consideration to predict GSs. In this study, two quantitative CT features and three qualitative CT features associated with GSs were selected to develop PSS. The AUC of the PSS for predicting GSs was 0.915, with an accuracy of 0.848, sensitivity of 0.843, and specificity of 0.850, which was similar to the results obtained by Wang et al, in which CT-analysis based machine learning model was used to differentiate GS from GIST (5). Contrary to a previously created model (1, 2, 5, 6) that might be time-consuming and difficult to use, this PSS provides an easy tool for radiologists to differentiate GSs from GISTs. In addition, no significant difference between the logistic regression model and PSS was observed, indicating the feasibility of PSS in the prediction of GSs. Therefore, the PSS proposed in this study can not only provide comprehensive information on GSs but also improve its utility in clinical decision-making.

Within the PSS, three qualitative CT features, including tumor location, growth patterns, and tumor surface ulceration, were identified as predictors for GSs. Contrary to most GISTs detected in the cardia and fundus (59.86%) of the gastric tissue, the gastric body is the most common site of GSs (76.47%), which was consistent with the results in previous studies (5, 15–17). Another predominant finding was a tendency toward exophytic or mixed growth patterns of GSs, which was different from GISTs and is also consistent with previous studies (1, 9, 18, 19). Furthermore, the incidence of tumor surface ulceration was significantly higher in GSs than in GISTs, which is in contrast to the lower incidence of surface ulceration in GSs in most previous reports (5, 20, 21) but is concordant with those of previous studies from Fujiwara et al. (22) and Wang et al. (16). A potential explanation for the results may be as follows: tumor surface ulceration may be attributed to the enlarging subepithelial tumor restricting circulation to the mucosa, making the tumor mucosal margin ischemic and more susceptible to damage by gastric acidity (2, 19). In this study, only very low- and low-risk GSTs were included, which had smaller tumor sizes. Thus, the incidence of tumor surface ulceration was significantly higher in GSs than in GSTs due to the tumor sizes.

Contrast-enhanced CT features play an important role in the differential diagnosis between GS and GIST. Based on the CT quantitative features, Value_TV_ was an independent indicator for GSs. In this study, GSs showed significantly higher Value_TV_ than GISTs (80 HU vs. 69 HU, *P*=0.015), and this result was similar to those of previous studies (5, 6). The lower Value_TV_ for GISTs may be due to the mismatch between the relatively slow speed of neovascularization and the fast speed of tumor growth and to the quick washout of intratumoral contrast agent in the portal phases for GISTs (5, 6, 23, 24). In contrast, GSs are relatively slow-growing tumors that are typically on par with those of neovascularization and exhibit a mild enhancement in the arterial phase with strengthening in the venous phase (4, 17, 25). These rationales could explain why the finding of Value_TV_ was significantly higher in GSs than in GISTs. Furthermore, TSR was introduced to eradicate confounding factors in CT attenuation values (10) and was also selected as another predictor for GSs. In this study, GSs had a significantly higher TSR than GISTs (0.65 vs. 0.56, *P*<0.001), which may be due to the rich vascular supply and gradual enhancement in the venous phase of GSs (17, 19).

This study has several limitations. First, although we included a relatively large cohort study with 51 GS patients, compared to most previous studies (5, 9, 17, 26–28), including more GS patients will make the results more reliable. Second, we only developed but did not validate the PSS due to the limits of the sample size. Independent validation will provide higher-level evidence for its clinical application. Third, only very low- or low-risk GISTs were considered in this study because distinguishing such GISTs from GSs is more difficult compared to the intermediate-to-high-risk of GISTs with large sizes and heterogeneous enhancement. However, this might be contaminated by memory bias. All risks of GISTs should be taken into account in future study. Fourthly, other gastric benign mesenchymal tumors such as glomus tumors, neurofibromas, ganglioneuromas, paragangliomas, and fibroblastic tumors are remarkably infrequently encountered. Therefore, we excluded these tumors from our differential diagnosis. Although gastric leiomyomas exhibit typical features in CT imaging characterized by slight enhancement, easily distinguishing them from GISTs. However, incorporating gastric leiomyomas and GSs into the study would increase the practicality of the findings in clinical settings. Future research will involve a comparative study of other gastric tumors.

## Conclusions

In conclusion, PSS based on Value_TV,_ TSR, tumor location, growth patterns, and tumor surface ulceration can provide an easy tool for radiologists to successfully differentiate GSs from GISTs before surgery and can complement the current diagnostic path for gastric tumors originating from the muscularis propria layer and exophytic growth tumors.

## Data availability statement

The original contributions presented in the study are included in the article/**Supplementary Material**. Further inquiries can be directed to the corresponding authors.

## Ethics statement

The studies involving human participants were reviewed and approved by Meizhou People’s Hospital. The ethics committee waived the requirement of written informed consent for participation.

## Author contributions

RZ, XGC, JF, and ZD proposed the study concepts, and then SIZ, XFC, and ZY designed the study. Data acquisition was performed by RH, SS, LH, YS, ZZ, and JY. SHZ conducted statistical analyses. All the authors participated in the data analysis and interpretation. All the authors read and approved the final manuscript.
