# Supplementary material for: Development of a CT image analysis-based scoring system to differentiate gastric schwannomas from gastrointestinal stromal tumors
Source: Front Oncol. 2023 Jun 28;13:1057979. doi: 10.3389/fonc.2023.1057979 (PMC10338089; doi:10.3389/fonc.2023.1057979)
Supplement: Supplementary file 1 [file DataSheet_1.docx]

**Supplementary Materials**

**Table of Contents:**

[**1. Gastrointestinal stromal tumor patients** **selection at Meizhou People's Hospital (Figure E1) 2**](#_Toc35425435)

[**2. CT imaging protocols and acquisition parameters (Table E1) 3**](#_Toc35425436)

[**3. Definition of radiological features (Table E2) 5**](#_Toc35425437)

[**4. One example of the evaluation of CT features (Figure E2) 7**](#_One_example_of)

**5.** [**Multivariate logistic regression analysis of radiological features (Table E3) 8**](#_Multivariate_logistic_regression)

# Gastrointestinal stromal tumor patients selection at Meizhou People's Hospital (Figure E1)


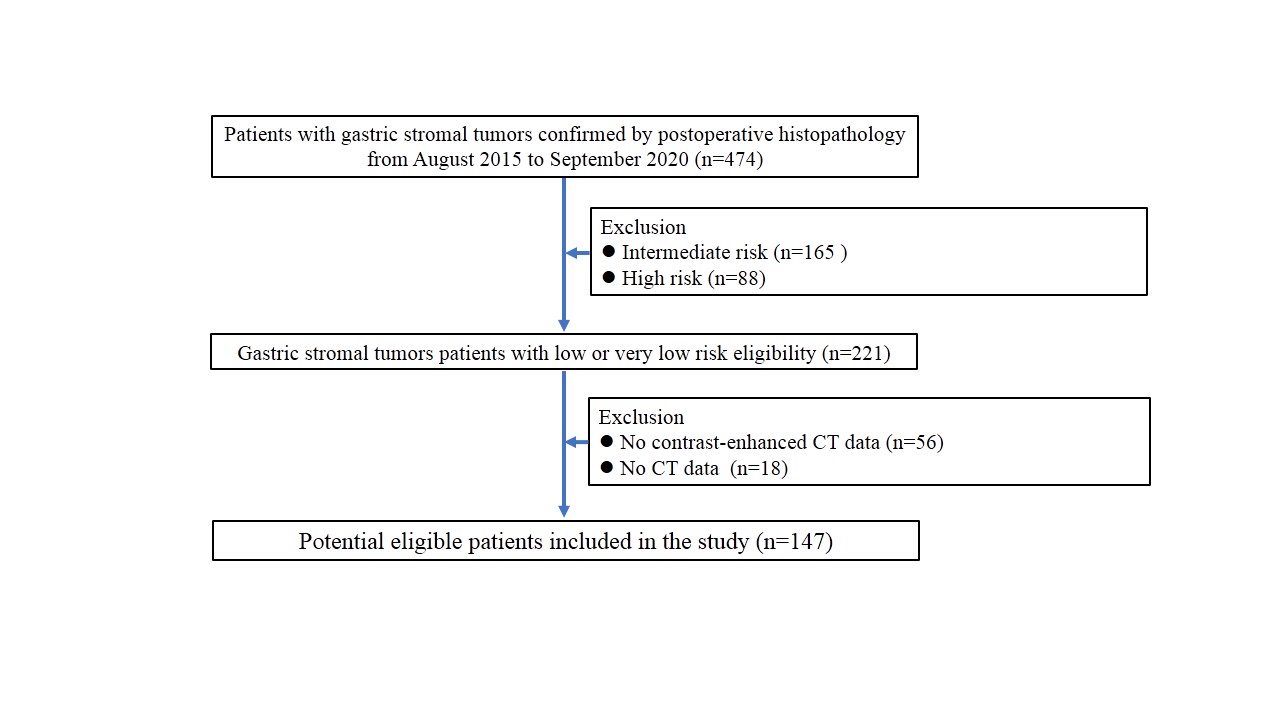


**Figure E1** Flowchart showing patients with gastrointestinal stromal tumor selection at Meizhou People's Hospital.

# CT imaging protocols and acquisition parameters (Table E1)

We selected patient images that were acquired on various models of multirow spiral CT scans obtained with GE, Siemens, Toshiba, and Philips scanners. Table E1 shows the CT imaging protocols and acquisition parameters of each medical institution.

| **Table E1**  CT imaging protocols and acquisition parameters of each medical institution | | | | | | |
| --- | --- | --- | --- | --- | --- | --- |
| CT imaging protocols and acquisition parameters | Meizhou People’s Hospital, Guangdong province | First Affiliated Hospital of Shantou University Medical College | Jiangmen Central Hospital, Guangdong province | The First Affiliated Hospital of Huzhou University | Daping Hospital, Army Medical University | Shantou Central Hospital, Guangdong province |
| CT scanner | Third-generation dual-source CT  (Force, Siemens) | 500-slice multidetect or spiral CT (Discovery HD750,GE) | 64-slice multidetector spiral CT (Aquilion one, Toshiba Medical Systems) | 64-slice multidetector spiral CT (Definition AS, Siemens) | 80-slice multidetector spiral CT (Brilliance iCT, PHILIPS) | 128-slice multidetector spiral CT (definition flash, Siemens) |
| Tube voltage (kV) | 100 | 120 | 120 | 120 | 120 | 120 |
| Automatic tube current (mA) | 180 (reference) | 200 | auto | auto | 200 | auto |
| Detector (mm) | 128 | 128 | 64 | 64 | 80 | 128 |
| Rotation time (s) | 0.25 | 0.9 | 0.5 | 0.5 | 0.5 | 0.75 |
| Slice thickness (mm) | 5.0 | 5.0 | 3.0 | 3.0 | 5.0 | 5.0 |
| Slice interval (mm) | 5.0 | 5.0 | 3.0 | 3.0 | 5.0 | 3.0 |
| Iterative Reconstruction technique | + | + | + | + | + | + |
| Reconstruction slice interval (mm) | 1.25 | 1.25 | 1.25 | 1.25 | 1.25 | 1.25 |
| Reconstruction slice thickness (mm) | 1.25 | 1.25 | 1.25 | 1.25 | 1.25 | 1.25 |
| Detector collimation | 0.6 | 0.625 | 0.625 | 0.625 | 0.625 | 0.625 |
| Pitch | 3.0 | 0.984 | 0.7 | 1.0 | 0.984 | 1.0 |
| Matrix | 512 × 512 | 512 × 512 | 512 × 512 | 512 × 512 | 512 × 512 | 512×512 |
| Field of view (mm) | 469×469 | 625×625 | 350×350 | 330×330 | 500×500 | 389×389 |
| Contrast medium | Omnipaque, 350 mg I/ml, GE | Ultravist, 300 mg/ml, BAYER | Ultravist, 300 mg/ml, BAYER | Iohexol, 300 mg I/ml, Yangzijiang Pharmaceutical Industry GROUP | Ultravist, 300 mg/ml, BAYER | Ultravist 370, 300 mg/ml, BAYER |
| Injection dose | 1.2～1.5 ml/kg | 1.2～1.5 ml/kg | 1.2～1.5 ml/kg | 1.5 ml/kg | 1.5 ml/kg | 1.5 ml/kg |
| Injection rate (ml/s) | 3.0–3.5 | 2.0–2.5 | 3.0–3.5 | 3.2–3.5 | 2.5–5.0 | 3.0 |
| Arterial phase (s) | 25–30 | 37 | 25–30 | 25 | 20–30 | 30 |
| Venous phase (s) | 60 | 81 | 60 | 50 | 55–65 | 60 |

# Definition of radiological features (Table E2)

Table E2 presents definitions of radiological features, which could help to understand each feature in the CT images and read them easily. These definitions are cited from the study by Ming-Yan He et al. in 2017[1].

**Table E2 Definition of radiological features**

| Radiological Features | Definition |
| --- | --- |
| Locations | The locations were classiﬁed as cardia and fundus, greater curvature of the body, lesser curvature of the body, and antrum according to the grading criteria for surgical difficulty |
| Tumor growth patterns |  |
| Endoluminal growth | Tumor located within the margin of the gastrointestinal structures |
| Exophytic growth | Intracavitary tumor extended beyond the margin of the gastrointestinal structures profile |
| Mixed growth | Tumor was located across the margins of gastrointestinal structures |
| Heterogeneous | Heterogeneous enhancement was deﬁned as areas of necrosis, cystic change, or varying degrees of enhancement (> 10 HU difference) during any contrast enhancement phase |
| Contour | The contour of the tumor was classiﬁed as round, quasi-circular, and lobulated |
| Margin |  |
| Well-defined | Tumor with smooth-edged margin |
| Ill-defined | Tumor with rough edged margin |
| Surface ulceration | Endoluminal growth tumor showed a focal tissue defect on the mucosal surface appearing as silt- or semielliptical-shaped lesions |
| Haemorrhage | A high-density region detected in the unenhanced CT scan with CT values of 60 to 80 HU |
| Cystic change | A low attenuation region on contrast-enhanced CT without obvious enhancement and CT attenuation value ≤20HU |
| Necrosis | A region with near-water density (0–10 HU) and without enhancement on contrast-enhanced CT |
| Calcification | A region with a density of 80 to 200 HU on a plain CT scan |

# One example of the evaluation of CT features (Figure E2)

Figure E2 presents one example of the evaluation of CT features on venous phase contrast-enhanced images obtained from a patient with a gastrointestinal stromal tumor.


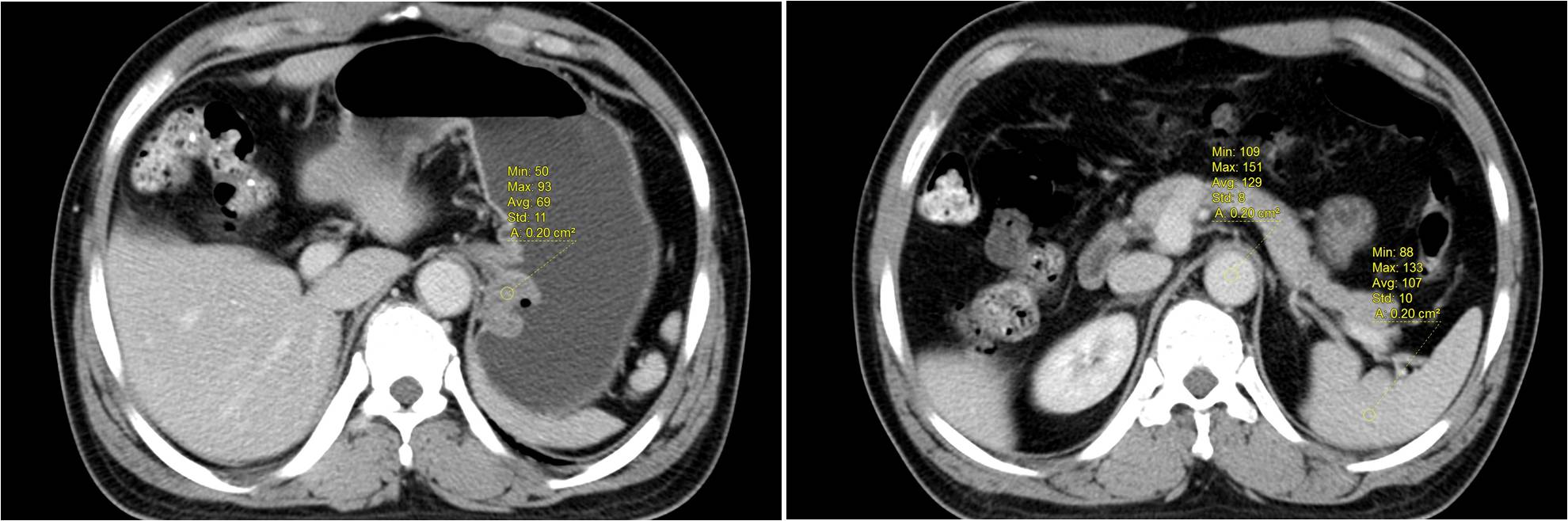


**Figure E2** A 54-year-old male with gastrointestinal stromal tumor. The axial venous phase contrast-enhanced CT image shows an endoluminal growth tumor in the cardia and fundus of the gastric tissue, with a well-defined margin and tumor surface ulceration. For CT quantitative parameter measurement, a circular region of interest (ROI) measuring 20 mm^2^ was manually placed on the maximal section with the greatest enhancement areas of tumors, avoiding obvious vessel structures, necrosis, calcification, ulceration, and cystic areas, and the mean CT attenuation value of the tumor in venous phase contrast-enhanced images (recorded as $\mathrm{Value}_{\mathrm{TV}}$) was 69 HU. Then, a ROI of the same size was placed in the homogeneous spleen parenchyma on the greatest cross-section of the spleen, and the mean CT attenuation value of the spleen in venous phase contrast-enhanced images (recorded as $\mathrm{Value}_{\mathrm{SV}}$) was 107 HU. Next, a ROI of the same size was placed in the same site of the spleen on the aorta, and the CT attenuation value of spleen in venous phase contrast-enhanced images (recorded as $\mathrm{Value}_{\mathrm{AV}}$) was 129 HU.

# Multivariate logistic regression analysis of the radiological features (Table E3)

| **Table E3** Multivariate logistic regression analysis of the radiological features | | |
| --- | --- | --- |
| Variables | Odds ratio (95% CI) | *P* value |
| $\mathrm{Value}_{\mathrm{TV}}$ | 0.96 (0.93–1.00) | **0.042** |
| TSR | 840.5 (16.67–42401.4) | **0.001** |
| Tumor Location |  |  |
| Cardia and fundus | Reference | Reference |
| Greater curvature | 15.33 (3.24–72.48) | **0.001** |
| Lesser curvature | 5.53 (1.10–27.86) | **0.038** |
| Antrum | 46.63 (6.62–328.45) | **<0.001** |
| Growth patterns |  |  |
| Endoluminal | Reference | Reference |
| Exophytic | 17.75 (5.01–62.96) | **<0.001** |
| Mixed | 6.33 (1.99–20.10) | **0.002** |
| Surface ulceration |  |  |
| Absent | Reference | Reference |
| Present | 6.83 (1.55–30.07) | **0.011** |

Reference

1. He MY, Zhang R, Peng Z, Li Y, Xu L, Jiang M, Li ZP, Feng ST: **Differentiation between gastrointestinal schwannomas and gastrointestinal stromal tumors by computed tomography**. *Oncol Lett* 2017, **13**(5):3746-3752.
